# Synthesis, Structure, Solid‐State NMR Spectroscopy, and Electronic Structures of the Phosphidotrielates Li_3_AlP_2_ and Li_3_GaP_2_

**DOI:** 10.1002/chem.202000482

**Published:** 2020-04-28

**Authors:** Tassilo M. F. Restle, Jasmin V. Dums, Gabriele Raudaschl‐Sieber, Thomas F. Fässler

**Affiliations:** ^1^ Department of Chemistry Chair for Inorganic Chemistry with Focus on New Materials Technische Universität München Lichtenbergstraße 4 85747 Garching Germany; ^2^ Department of Chemistry Chair of Inorganic and Metal-Organic Chemistry Technische Universität München Lichtenbergstraße 4 85747 Garching Germany

**Keywords:** aluminum, gallium, NMR spectroscopy, phosphorous, semiconductors

## Abstract

The lithium phosphidoaluminate Li_9_AlP_4_ represents a promising new compound with a high lithium ion mobility. This triggered the search for new members in the family of lithium phosphidotrielates, and the novel compounds Li_3_AlP_2_ and Li_3_GaP_2_, obtained directly from the elements via ball milling and subsequent annealing, are reported here. It was unexpectedly found through band structure calculations that Li_3_AlP_2_ and Li_3_GaP_2_ are direct band gap semiconductors with band gaps of 3.1 and 2.8 eV, respectively. Rietveld analyses reveal that both compounds crystallize isotypically in the orthorhombic space group *Cmce* (no. 64) with lattice parameters of *a=*11.5138(2), *b=*11.7634(2) and *c=*5.8202(1) Å for Li_3_AlP_2_, and *a=*11.5839(2), *b=*11.7809(2) and *c=*5.8129(2) Å for Li_3_GaP_2_. The crystal structures feature *Tr*P_4_ (*Tr*=Al, Ga) corner‐ and edge‐sharing tetrahedra, forming two‐dimensional ${}_{\infty }{}^{2}\left[{TrP_{2}}^{3-}\right]$ layers. The lithium atoms are located between and inside these layers. The crystal structures were confirmed by MAS‐NMR spectroscopy.

## Graphical abstract

**Layer structure in phosphidoaluminates and ‐gallates**: The two new isotopic structures of Li_3_AlP_2_ and Li_3_GaP_2_ exhibit layers of corner‐ and edge‐sharing AlP_4_/GaP_4_ tetrahedra. The compounds are easily accessible through ball milling of the elements and are both semiconductors with a direct band gap of 3.1 and 2.8 eV, respectively.

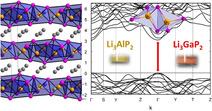

## Introduction

Lithium ion solid electrolytes have been intensively studied for years due to the promising enhanced safety and electrochemical performances of all‐solid‐state‐batteries.^1^, ^2^, ^3^ Thus, many new materials with potentially high lithium ion conductivity have been discussed in the literature.^3^, ^4^, ^5^, ^6^, ^7^ Recently, with Li_14_SiP_6_, Li_8_SiP_4_ and *α*/*β*‐Li_8_GeP_4_, we introduced group 14 phosphide‐based lithium ion conductors, which achieve ionic conductivities up to 1 mS cm^−1^.^8^, ^9^, ^10^ Their structures are built by group 14 phosphorous tetrahedra [*Tt*P_4_]^8−^ (*Tt*=Si, Ge). In the case of Li_8_SiP_4_ and *α*/*β*‐Li_8_GeP_4_, isolated [*Tt*P_4_]^8−^ tetrahedra occur. At lower Li contents we found that the tetrahedra are connected in different ways and form dimers as in Li_10_Si_2_P_6_, two‐dimensional slabs as in Li_3_Si_3_P_7_, or three‐dimensional networks as in Li_2_SiP_2_.^8^, ^11^ Interestingly, the phases Li_8_SiP_4_, Li_5_SiP_3_ (=Li_10_Si_2_P_6_), Li_2_SiP_2_, and LiSi_2_P_3_ are connected by a formal reduction of the formula by units of Li_3_P.^11^ A lower Li_3_P content leads to a higher connectivity of the tetrahedra.

Compared to the related sulfide‐based lithium ion conductors,^3^, ^6^, ^7^, ^12^ the anionic substructure of phosphido‐based conductors carry one additional charge (formal “P^3−^” versus a formal “S^2−^”), and thus the Li content that is required for charge balance is higher. Recently, we expanded this concept of highly charged tetrahedra to lithium phosphidoaluminates by replacing the central group 14 metal by aluminium.^13^

Li_9_AlP_4_ contains highly charged [*Tr*P_4_]^9−^ tetrahedra and reaches high ionic conductivities of ≈3.0 mS cm at room temperature. Besides this first report of a structurally characterized lithium phosphidoaluminate, another compound of the composition Li_3_AlP_2_ was mentioned already in 1952 and described with an orthorhombic distorted CaF_2_‐type structure, in which the phosphorus atoms form a distorted cubic close packing, although without reliable crystallographic data.^14^ Two years later, the corresponding gallium compound Li_3_GaP_2_ was also postulated.^15^ Despite the poorly characterized structure model, quantum‐chemical calculations of Li_3_AlP_2_ and Li_3_GaP_2_ were performed, anticipating the model of vertex‐sharing AlP_4_ tetrahedra.^16^, ^17^, ^18^ As for lithium phosphidotetrelates, lithium phosphidoaluminates can also be connected on a line in a Gibbs composition triangle (Finetti diagram). Li_3_AlP_2_ is located on the line in the phase system Li‐Al‐P connecting Li_3_P and AlP (Figure S7, Supporting Information) by reducing Li_9_AlP_4_ by two units of Li_3_P (Li_3_AlP_2_=Li_9_AlP_4_−2×Li_3_P). Assuming a charge balanced valence compound, the degree of connectivity of the AlP_4_ tetrahedra in Li_3_AlP_2_ must be higher, and isolated tetrahedra as observed in Li_9_AlP_4_ cannot occur.

Here we report on the synthesis and structural characterization of Li_3_AlP_2_ and Li_3_GaP_2_ by a simple ball milling approach. Both compounds are characterized by Rietveld analysis and MAS‐NMR spectroscopy. In addition, electronic band structure calculations are discussed.

## Experimental Section

Syntheses and sample preparation and all sample manipulations were carried out inside an argon‐filled glove box (MBraun, *p*(H_2_O), *p*(O_2_)<0.1 ppm). Lithium (Li, rods, Rockwood Lithium, >99 %) was cleaned of oxide layers prior to use. Aluminium (Al, granules, ChemPur, 99,99 %), gallium (Ga, pieces, ChemPur, 99,99 %) and phosphorus (P, powder, Sigma–Aldrich, 97 %) were used without any further purification.

**Figure 1:**
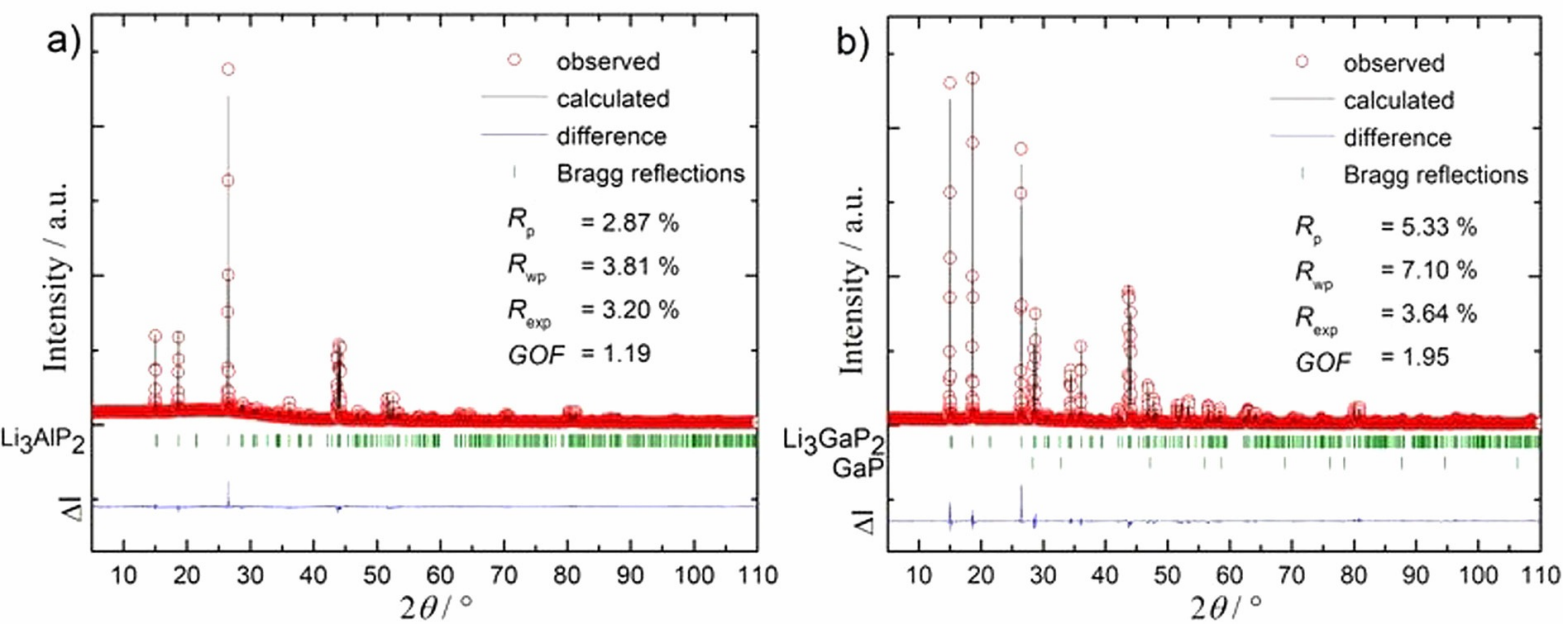
X‐ray powder diffractograms and results from the Rietveld analysis of Li_3_ *Tr*P_2_. The red, black and blue lines indicate the observed and the calculated intensities, and the difference between both, respectively. a) Rietveld analysis of Li_3_AlP_2_. Bragg positions are given in green dashes. b) Rietveld analysis of Li_3_GaP_2_. Bragg positions for Li_3_GaP_2_ and GaP are given in green dashes. The ratio of Li_3_GaP_2_ to GaP is 98.58(3): 1.42(5) wt. *%*.

**Synthesis of Li_3_** ***Tr*** **P_2_ (*Tr*=Al, Ga)**: Li_3_ *Tr*P_2_ was synthesized from the elements via ball milling and subsequent annealing. **Li_3_AlP_2_**: Lithium (388.0 mg, 55.3 mmol, 3 equiv), aluminium (498.1 mg, 18.5 mmol, 1 equiv) and phosphorus (1178.0 mg, 36.9 mmol, 2 equiv) were loaded in a WC milling set (50 mL jar, 3 balls with a diameter of 1.5 cm) and ball milled using a Retsch PM100 Planetary Ball Mill for 36 h at 350 rpm with resting periods (for 3 min every 10 min). **Li_3_GaP_2_**: Lithium (350.8 mg, 50.0 mmol, 3 equiv), gallium, (1163.0 mg, 16.7 mmol, 1 equiv) and phosphorus (1065.1 mg, 33.4 mmol, 2 equiv) were transferred to a WC milling set (45 mL jar, 7 balls with a diameter of 1.5 cm) and ball milled using a Fritsch Pulverisette 6 for 18 h at 350 rpm with resting periods (for 5 min every 10 min). For Li_3_AlP_2_ an ochre, and for Li_3_GaP_2_ a red powder is obtained. The powders were pressed into pellets with a diameter of 13 mm for 30 sec. at 5 t using a hydraulic press (Specac Atlas 15T). The fragmented pellets were filled into niobium ampoules which were sealed in an electric arc furnace (Edmund Bühler MAM1). The sealed ampules were enclosed in evacuated silica reaction containers and heated in a tube furnace (HTM Reetz Loba) up to 700 °C at 5 K min^−1^, dwelled for 24 h and subsequently cooled at 0.5 K min^−1^ to room temperature. After grinding of the pellets, a yellow‐ochre powder is obtained for Li_3_AlP_2_ and a brick‐red powder for Li_3_GaP_2_ (see Figure S3 in Supporting Information). Li_3_AlP_2_ was obtained phase pure, whereas the sample of Li_3_GaP_2_ showed a few reflections of GaP with low intensity (see Figure 1).

In an alternative synthesis, Li_3_ *Tr*P_2_ is obtained by reacting stoichiometric amounts of the elements in a tantalum ampule. For Li_3_AlP_2_, lithium (96.0 mg, 13.7 mmol, 3.0 equiv), aluminium (123.0 mg, 4.6 mmol, 1.0 equiv) and phosphorus (291.0 mg, 9.1 mmol, 2.0 equiv), and for Li_3_GaP_2_, lithium (34.5 mg, 4.9 mmol, 3.0 equiv), gallium (115.5 mg, 1.7 mmol, 1.0 equiv) and phosphorus (104.7 mg, 3.3 mmol, 2.0 equiv) were filled into a tantalum ampule. The ampules were sealed in an electric arc furnace (Edmund Bühler MAM1), enclosed in a quartz reaction container under vacuum and subsequently heated at 5 K min^−1^ up to 550 °C, dwelled for seven days, and then cooled at 0.075 K min^1^ to room temperature in a tube furnace (HTM Reetz Loba 1200‐42‐600‐1‐OW with a EUROTHERM S 14083 temperature controller), yielding Li_3_AlP_2_ (light ochre) and Li_3_GaP_2_ (red). In contrast to the ball mill synthesis, both products contain more impurities, with a few unknown reflections, accompanied by reflections of TaP (Li_3_AlP_2_) and GaP (Li_3_GaP_2_) (see Figure S4 and S5).

**Powder X‐ray diffraction**: For powder X‐ray diffraction (PXRD) measurements, the samples were grounded in an agate mortar and sealed inside 0.3 mm glass capillaries. PXRD measurements were performed at room temperature on a STOE Stadi P diffractometer equipped with a Ge(1 1 1) monochromator for Cu${}_{K\alpha {}_{1}}$ radiation (*λ*=1.54056 Å) and a Dectris MYTHEN DCS 1 K solid‐state detector. The raw powder data were processed with the software package WinXPOW.^19^

**Structure determination and Rietveld refinement**: The structures of Li_3_ *Tr*P_2_ were determined by Rietveld refinements of the powder X‐ray diffraction data using JANA2006.^20^ The initial structure model was gained using the subprogram Superflip.^21^ The space group *Cmce* (no. 64) and the cell parameters were determined with the subprogram system evaluation of WinXPOW.^19^ All cell parameters, all atom positions and the isotropic displacement parameters of *Tr* and P were refined freely. The isotropic displacement parameters of all Li atoms were refined coupled in Li_3_AlP_2_ and uncoupled in Li_3_GaP_2_.

CCDC 1979150 (Al) and 1979151 (Ga) contain the supplementary crystallographic data for this paper. These data are provided free of charge by The Cambridge Crystallographic Data Centre through the CCDC/FIZ Karlsruhe deposition service.

**Energy‐dispersive X‐ray spectroscopy (EDX)**: Pieces of Li_3_ *Tr*P_2_ were measured on a Hitachi TM‐1000 Tabletop (15 kV) scanning electron microscope equipped with an energy dispersive X‐ray analyser (SWIFT‐ED‐TM). The samples were mounted onto an aluminium stub using graphite tape. To exclude aluminium impurities of the stub in the EDX spectra, measurements of the graphite tape on the aluminium stub without the samples were carried out, and no aluminium signal was registered. All samples were measured three times, and the values were averaged.

**Differential scanning calorimetry (DSC)**: For thermal analysis samples of Li_3_ *Tr*P_2_ were sealed in niobium ampules and measured on a DSC instrument (Netzsch, DSC 404 Pegasus) under a constant gas flow of 75 mL min^−1^. The samples were heated to 750 °C and then cooled to 150 °C twice at a rate of 10 °C min^−1^. For the determination of the onset temperatures of the DSC signals, the PROTEUS Thermal Analysis software was used.^22^

**Impedance spectroscopy**: The electrochemical impedance spectroscopy for Li_3_ *Tr*P_2_ was performed in an in‐house designed cell. The detailed setup and procedure are described in Restle et al.^13^ Impedance spectra were recorded on a Bio‐Logic potentiostat (SP‐300) in a frequency range from 7 MHz to 50 mHz at a potentiostatic excitation of ±50 mV. Data were treated using the software EC‐Lab (V 11.27). The measurements were performed in an Ar‐filled glove box at 26 °C.

**NMR spectroscopy**: Magic‐angle spinning (MAS) NMR spectra have been recorded on a Bruker Avance 300 NMR device operating at 7.04 T in a 4 mm ZrO_2_ rotor. The resonance frequencies of the nuclei are 44.17, 78.21, 91.53, and 121.46 MHz for ^6^Li, ^27^Al, ^71^Ga, and ^31^P, respectively. The rotational frequency was set to 15 kHz for all nuclei. The MAS spectra have been obtained at room temperature with relaxation delays of 10 s (^6^Li), 2 s (^27^Al), 2 s (^71^Ga), and 30 s (^31^P), and 800 scans (^6^Li), 280 scans (^27^Al), 200 scans (^71^Ga), and 720 scans (^31^P). All ^6^Li spectra were referenced to LiCl (1 m, aq) and LiCl (s) with chemical shifts of 0.0 ppm and −1.15 ppm, respectively. The ^27^Al spectrum is referred to aluminium nitrate nonahydrate (s) with a chemical shift of −0.54 ppm with reference to Al(H_2_O)_6_^3+^ in aqueous solution. The ^71^Ga spectrum is referred to gallium nitrate monohydrate (1 m, aq) with a chemical shift of 0 ppm. The ^31^P spectra were referred to ammonium dihydrogen phosphate (s) with a chemical shift of 1.11 ppm with reference to concentrated H_3_PO_4_. All spectra were recorded using single‐pulse excitation.

**Electronic structure calculations**: The computational analysis for the structures Li_3_AlP_2_ and Li_3_GaP_2_ was performed using the Crystal17 program package and hybrid density functional methods.^23^, ^24^ A hybrid exchange correlation functional after Perdew, Burke and Ernzerhof (PBE0)^25^, ^26^ and triple‐zeta valence + polarization level basis sets derived from the Karlsruhe basis sets for the elements Li, Al, Ga, and P were applied (further details are in the Supporting Information).^27^, ^28^, ^29^ The starting geometry was taken from the experimental findings, and all structures were fully optimized within the constraints imposed by the space group symmetry. Band structures and density of states (DOS) were calculated for both structures. The nature of a stationary point on the potential energy surface was confirmed to be a minimum by a frequency calculation for each compound at Γ‐point. No imaginary frequencies were observed. For data processing and visualization Jmol was used.^30^

## Results and Discussion

### Synthesis and characterization of Li_3_ *Tr*P_2_

Phase‐pure Li_3_AlP_2_ and almost phase‐pure Li_3_GaP_2_ were synthesized from the elements via a two steps procedure. Firstly, stoichiometric amounts of Li, *Tr* and P were ball milled resulting in reactive mixtures which showed the most intense reflections with large half width of the corresponding compound in the X‐ray powder diffractogram (see Figures S1 and S2 in Supporting Information). Subsequently, pellets of the reactive mixtures were annealed in niobium ampules at 700 °C for one day, yielding phase‐pure Li_3_AlP_2_ and Li_3_GaP_2_, which contained small amounts of GaP as a side phase (see Figure 1). Powdered Li_3_AlP_2_ is yellow‐ochre, powdered Li_3_GaP_2_ is brick‐red (see Figure S3). Energy dispersive X‐ray spectroscopy (EDX) investigations of the products show the absence of W and Nb and are in very good accordance with the Al/P und Ga/P ratios used in syntheses (see Table S1). Li_3_AlP_2_ and Li_3_GaP_2_ can also be synthesized by heating stoichiometric amounts of the respective elements at 550 °C for one week. However, an unknown phase accompanied by TaP remains as impurity in Li_3_AlP_2_, whereas the sample of Li_3_GaP_2_ contains GaP plus another unknown phase (see Figures S4 and S5). Due to the good quality of the powder diffractograms the structures of Li_3_AlP_2_ and Li_3_GaP_2_ could be solved and refined from the powder X‐ray diffraction data. The results from the Rietveld refinement are shown in Figure 1, and parameters are listed in Table 1.

**Table 1:** Crystallographic data of Li_3_AlP_2_ and Li_3_GaP_2_ obtained by Rietveld analysis of the powder diffraction data.

empirical formula	Li_3_AlP_2_	Li_3_GaP_2_
formula weight [g mol^−1^]	109.75	152.49
*T* [K]	300	300
radiation wavelength	*λ*=1.5406 Å	*λ*=1.5406 Å
Colour	yellow ochre	brick red
crystal system	orthorhombic	orthorhombic
space group	*Cmce* (no. 64)	*Cmce* (no. 64)
unit cell dimension		
*a* [Å]	11.5138(2)	11.5839(2)
*b* [Å]	11.7634(2)	11.7809(2)
*c* [Å]	5.8202(1)	5.8129(2)
*V* [Å^3^]	788.29(2)	793.28(2)
*Z*	2	2
*ρ* (calc.) [g cm^−3^]	1.8496	2.5536
*Θ* range [°]	5.062–110.002	5.029–109.999
*R*_p_	0.0287	0.0533
*R*_wp_	0.0381	0.0710
*R*_exp_	0.0320	0.0364
goodness‐of‐fit	1.19	1.95
depository no.	1979150	1979151

**Figure 2:**
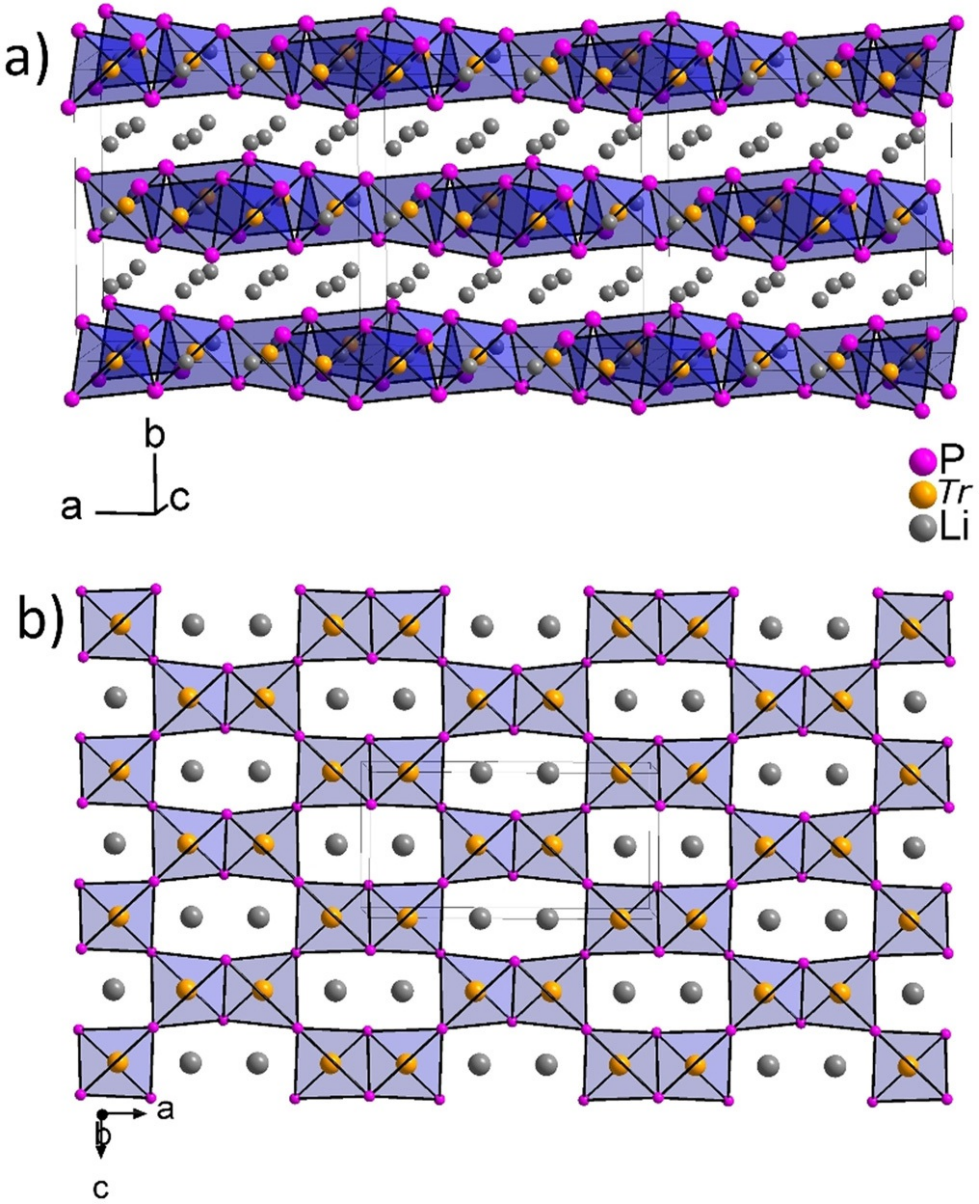
a) 3*×*1*×*1 Unit cell of the crystal structure of Li_3_ *Tr*P_2_. b) 3*×*3 on top view of one layer in Li_3_TrP_2_ in b direction. Li, *Tr* and P are depicted in grey, orange and purple, respectively (displacement ellipsoids are set at 90 *%* at room temperature).

Li_3_AlP_2_ and Li_3_GaP_2_ crystallize in the orthorhombic space group *Cmce* (no. 64) with five independent crystallographic positions (P1, P2, *Tr*1, Li1, and Li2) (Table S2). Compared to the earlier reported cell (*a*=11.47, *b*=11.61 and *c*=11.73 Å), which corresponds to a 2×2×2 orthorhombic distorted supercell of the anti‐CaF_2_ structure type,^14^ we observe a corresponding 2×2×1 orthorhombic supercell. A 3×1×1 unit cell of the orthorhombic crystal structure is displayed in Figure 2 a. The crystal structure is built up by an orthorhombic distorted cubic close packing of P atoms. The *Tr* atoms occupy one quarter of the tetrahedral voids, forming AlP_4_ tetrahedra. The occupation occurs in a fully ordered manner and is found only in every second layer. Within the layer the *Tr* atoms occupy 50 % of the tetrahedral voids. Pairs of the resulting AlP_4_ tetrahedra are connected by sharing edges through P1, and the resulting dimers share corners through P2, resulting in a two‐dimensional ${}_{\infty }{}^{2}\left[{TrP_{2}}^{3-}\right]\;$ layer (Figure 2 b). All remaining tetrahedral voids based on a *ccp* packing of P atoms are occupied with lithium, whereby Li1 is located within the ${}_{\infty }{}^{2}\left[{TrP_{2}}^{3-}\right]\;$ layers, and Li2 occupies the tetrahedral voids between the layers. The stacking sequence of the ${}_{\infty }{}^{2}\left[{TrP_{2}}^{3-}\right]$ layers is ABAB, as shown in Figure S6, in which the edge‐sharing Al_2_P_6_ dimers of the adjacent layers are located above the neighbouring tetrahedral sites, which are occupied by Li (shift along *a* by *a*/2). Li_3_ *Tr*P_2_ has a similar structure as LiNa_2_AlP_2_, in which exclusively Na atoms are located between, and Li atoms within the ${}_{\infty }{}^{2}\left[{AlP_{2}}^{3-}\right]$ layers, resulting in a larger separation of the layers (longer *b*‐axis with 13.592(3) Å in LiNa_2_AlP_2_ compared to 11.7634(2) Å in Li_3_AlP_2_).^31^

The bond lengths in the *Tr*P_4_ tetrahedra are in the narrow range between 2.398(3) and 2.410(3) Å for the Al1−P1 and Al1−P2 distances, respectively, and between 2.404(2) and 2.419(2) Å for the Ga1−P1 and Ga1−P2 distances, respectively. As expected, the Al−P bonds are slightly shorter than the Ga−P bonds. The Al−P bond lengths are very similar to those in LiNa_2_AlP_2_ (2.410(3)–2.426(3) Å) and are in the range of other known compounds with strong Al−P interactions like in AlP (2.360 Å), Na_3_AlP_2_ (2.376(4) Å) or in Sr_3_Al_2_P_4_ (2.377(3)–2.417(2) Å) and weaker Al−P interactions like in Li_9_AlP_4_ (2.423(2)–2.434(1) Å).^13^, ^31^, ^32^, ^33^, ^34^ In the case of *Tr*=Ga, the Ga−P bonds are longer than in GaP (2.3601(1) Å) and slightly shorter than in Ba_3_GaP_3_ (2.43(1) Å), where [Ga_2_P_6_]^6−^ dimers occur.^32^ Due to the edge‐ und corner‐sharing situation of the *Tr*P_4_ tetrahedra, these *Tr*P_4_ units are distorted. This distortion is exemplified by the deviation of the P‐Al‐P angles between 101.70(1)° and 111.57(1)° and of the P‐Ga‐P angles between 100.6(1)° and 112.18(1)° from the ideal tetrahedral angle of 109.47°. The Li‐P bond lengths in Li_3_AlP_2_ range from 2.51(1) to 2.61(1) Å and from 2.50(1) to 2.65(1) Å in Li_3_GaP_2_. Overall, these distances are in good agreement compared to other binary or ternary phases containing Li and P.^8^, ^9^, ^11^ Considering three positively charged lithium atoms and the threefold negatively charged ${}_{\infty }{}^{2}\left[{TrP_{2}}^{3-}\right]\;$ 2D layer, Li_3_ *Tr*P_2_ can be written as an electronically balanced formula (Li^+^)_3_ ${}_{\infty }{}^{2}\left[{TrP_{2}}^{3-}\right]$ .

The lattice parameters of Li_3_AlP_2_ and Li_3_GaP_2_ vary only slightly in the *b*‐ and *c*‐axes, whereas the *a*‐axes are significantly different. Hence, the substitution of the aluminium atoms within the tetrahedra by the larger gallium atoms (ionic radii 0.53 for Al^3+^ and 0.61 Å Ga^3+^ by Shannon and Prewitt)^35^ leads to an anisotropic enlargement of the ${}_{\infty }{}^{2}\left[{TrP_{2}}^{3-}\right]\;$ 2D layer due to the fact that in *a*‐direction the tetrahedra share edges and corners, whereas in *c‐*direction they are exclusively connected by corners (as shown in Figure 2 b and Figure S6).

The size of the alkali‐metal atom has a strong impact on the connectivity of the AlP_4_ tetrahedra. In LiNa_2_AlP_2_, the identical 2D ${}_{\infty }{}^{2}\left[{AlP_{2}}^{3-}\right]$ layers of AlP_4_ tetrahedra exist like in Li_3_AlP_2_, although with larger distances between the layers due to the larger Na ions that are located between the layers. The same applies to LiK_2_AlP_2_. Interestingly, in Na_3_AlP_2_ a one‐dimensional ${}_{\infty }{}^{1}\left[{AlP_{2}}^{3-}\right]$ chain with exclusively edge‐sharing tetrahedra is observed. The reason might be that the large Na atoms do not allow a filling of the tetrahedra. More space is provided, when the Na atoms are located between the chains.^33^ In the case of Cs_3_AlP_2_ a different structure is adopted.^36^ The Al atoms are coordinated in a trigonal planar manner by phosphorus atoms, leading to dimers of edge‐sharing triangles. In the case of related gallium compounds, only ternary alkali metal phosphidogallates with trigonal planar GaP_3_ triangles are reported (Na_6_GaP_3_, K_2_GaP_2_, Rb_3_GaP_2_, Cs_6_Ga_2_P_4_),^37^, ^38^, ^39^, ^40^ and Li_3_GaP_2_ represents the first ternary alkali metal‐based phosphidogallate with gallium in a tetrahedral coordination environment. Further, in quaternary mixed‐alkali metal phosphidogallates, such GaP_4_ tetrahedra already exist, for example, in K_2_NaGaP_2_ and Cs_2_NaGaP_2_.^41^, ^42^ As observed for phosphidoaluminate derivatives with larger alkali metals like Na_3_AlP_2_, in K_2_NaGaP_2_ and Cs_2_NaGaP_2_, the GaP_4_ tetrahedra are arranged in edge‐sharing 1D chains. Related alkaline earth metal phosphidotrielates contain the same polyanion ${}_{\infty }{}^{2}\left[{TrP_{2}}^{3-}\right]$ . Formally three Li ions are replaced by one and a half alkaline earth metal, such as in Ca_3_Al_2_P_4_, Ca_3_Ga_2_P_4_, Sr_3_Ga_2_P_4_ and Ba_3_Al_2_P_4_.^34^, ^43^ A structural change depending on the size of the alkaline earth metal atom can also be observed in these species. In the case of the smaller Ca and Sr atoms the structures contain distorted 2D layers of edge‐ and corner‐sharing *Tr*P_4_ tetrahedra. However, in Ba_3_Al_2_P_4_, the larger Ba atoms lead to a segregation into twisted chains with only edge‐sharing AlP_4_ tetrahedra.

### Differential scanning calorimetry

DSC measurements of both compounds were performed (Figures S9 and S10) and show that Li_3_AlP_2_ is stable up to 750 °C, whereas Li_3_GaP_2_ is stable only up to about 710 °C. Above this temperature Li_3_GaP_2_ might melt or decompose into other unknown phases, as also supported by the PXRD data after the measurement (Figures S11 and S12).

### Impedance spectroscopy

The Nyquist‐plots for Li_3_AlP_2_ and Li_3_GaP_2_ are shown in Figure S17 and Figure S18. The Nyquist‐plots display only the behaviour of a capacitor. Hence, no lithium diffusion was observed by electrochemical impedance spectroscopy.

### MAS‐NMR spectroscopy

**Figure 3:**
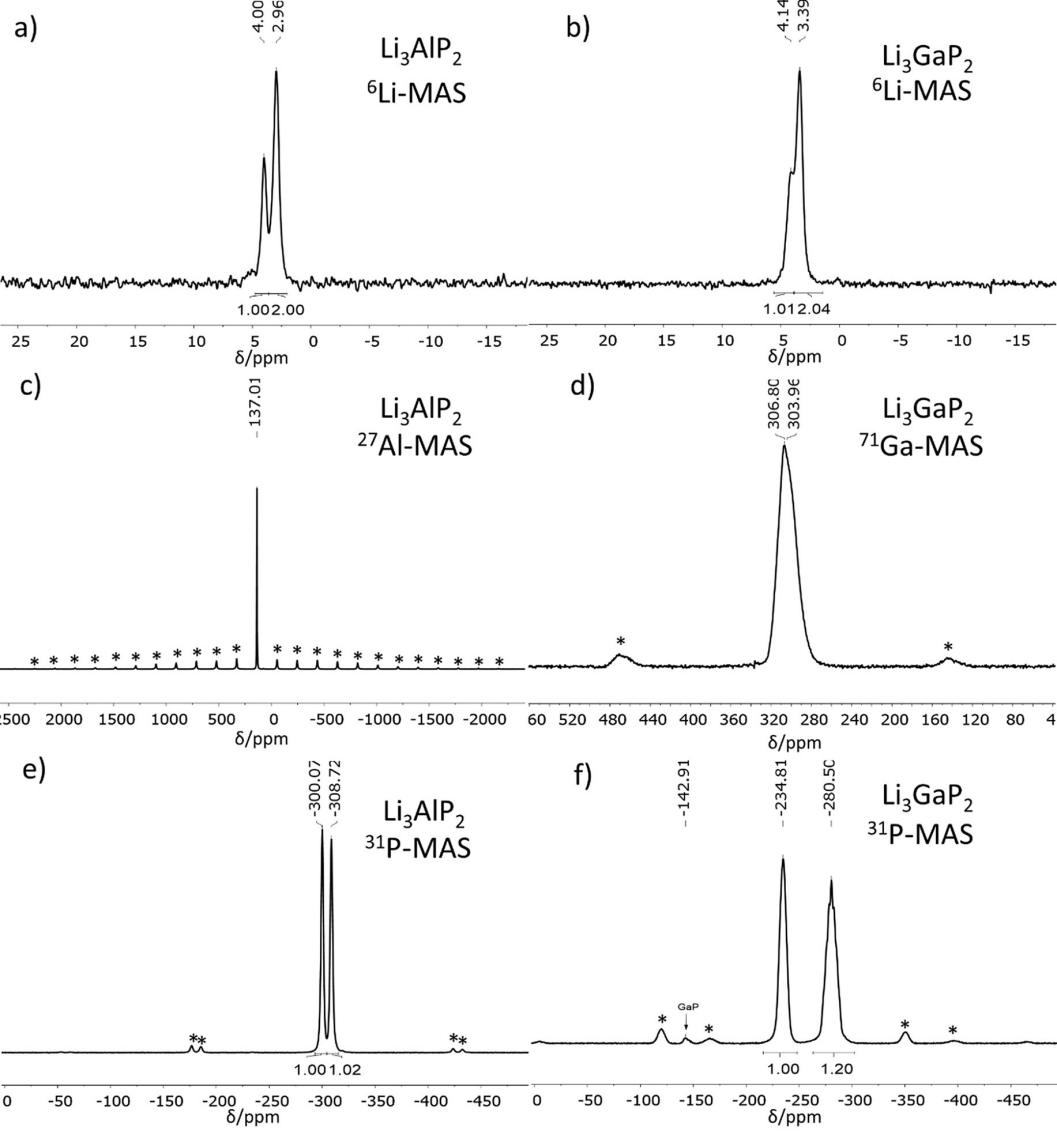
^6^Li (a, b), ^27^Al (c), ^71^Ga (d) and ^31^P (e, f) MAS‐NMR spectra of Li_3_AlP_2_ (left) and Li_3_GaP_2_ (right). Spinning sidebands are marked with an asterisk.

For Li_3_AlP_2_ and Li_3_GaP_2_, ^6^Li, ^27^Al, ^71^Ga, and ^31^P MAS‐NMR measurements were performed (see Figure 3). In agreement with the crystallographic multiplicity, two independent ^6^Li signals occur in the expected ratio of 1:2 (4.00 and 2.96 ppm in Li_3_AlP_2_ and 4.14 and 3.39 ppm in Li_3_GaP_2_). The lithium atoms inside the ${}_{\infty }{}^{2}\left[{TrP_{2}}^{3-}\right]$ layers are shifted more downfield than the others. In comparison to the signals of the aluminium phase, the resonances of both lithium signals in the gallium phase are shifted to lower fields. Hence, the layer itself and the more electronegative metal gallium lead to a higher deshielding of the signals. For both compounds the chemical shift of the Li atoms are in the same range as those for related phosphidosilicates like Li_8_SiP_4_ and Li_3_Si_3_P_7_.^8^, ^11^ The ^27^Al, respectively ^71^Ga NMR spectra show only one signal in accordance with the crystal structure. The Al shift of 137 ppm utterly fits to the one of tetrahedral aluminium phosphines in solution and matches almost perfectly to the tetrahedrally coordinated Al in AlP (142 ppm).^44^, ^45^ The chemical shift of 304 ppm of Ga also is in good agreement with the tetrahedral environment of Ga in GaP (307 ppm).^45^ The shape of the ^71^Ga signal is slightly asymmetric due to small GaP impurities at 307 ppm. Li_3_AlP_2_ shows two singlets in the ^31^P MAS‐NMR spectrum. Both signals can be integrated with a value of one. Their chemical shifts are in the range of isolated P^3−^ in Li_3_P and tetrahedrally coordinated P in Li_8_SiP_4_.^8^, ^46^ For Li_3_GaP_2_ two main signals occur with almost the same integrated intensity. The ^31^P signals are shifted slightly more to lower fields than in Li_3_AlP_2_. The small signal at −143 ppm can be assigned to GaP.^47^ Summing up, the NMR measurements are in very good agreement with the crystal structure evaluation on the basis of the Rietveld analyses.

### Electronic structures

**Figure 4:**
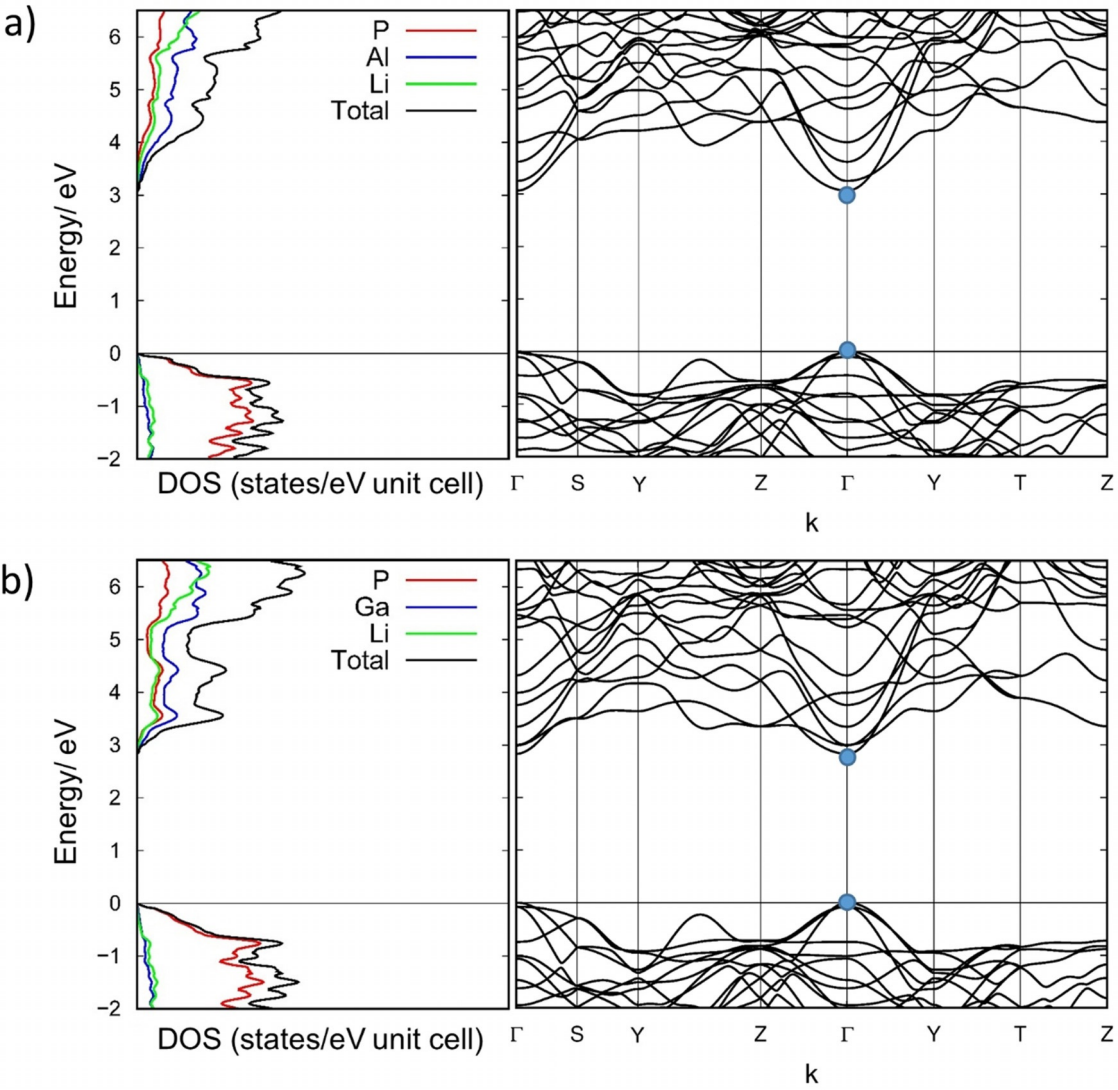
Density of states (DOS) (left) and Band structure (right) of a) Li_3_AlP_2_ exhibiting a direct band gap of 3.1 eV and b) Li_3_GaP_2_ exhibiting a direct band gap of 2.8 eV. The Fermi level is located at 0 eV. Blue points in the band structure diagram correspond to the top and bottom of the valence and conduction band, respectively.

For Li_3_AlP_2_ and Li_3_GaP_2_ a computational analysis was carried out at a DFT‐PBE0/TZVP level of theory. The optimized structure for Li_3_AlP_2_ and Li_3_GaP_2_ exhibit a maximum deviation of 1.95 % for all parameters and average atomic distances, which reassures the experimental findings (Table 2). Band structure calculations show that both compounds are semiconductors with direct band gaps (Figure 4). Due to the usage of hybrid functional the calculated band gaps are typically in good agreement with the experiment. The calculated band gap of Li_3_GaP_2_ of 2.8 eV is significantly smaller than the one of Li_3_AlP_2_ (3.1 eV). This divergence is in accordance with the two different colours of the phases: Li_3_AlP_2_ with a larger band gap is of yellow‐ochre colour, whereas Li_3_GaP_2_ with a smaller band gap is brick red. The densities of states reveal that the contribution of phosphorus is the highest at the valence band maximum, whereas in the conduction band minimum aluminium and gallium have the highest contributions. The calculated band structure is typical of a direct band gap semiconductor.

**Table 2:** Atomic distances (*d*) and cell parameters *a*, *b* and *c* for Li_3_AlP_2_ and Li_3_GaP_2_ as well as deviation (Δ*d*) from experimental data in percentages.

	Li_3_AlP_2_ *d* [Å]	Li_3_AlP_2_ Δ*d* [%]	Li_3_GaP_2_ *d* [Å]	Li_3_GaP_2_ Δ*d* [%]
*a*	11.5388	0.22	11.5910	0.02
*b*	11.7560	0.06	11.7834	0.02
*c*	5.8267	0.11	5.8289	0.24
Av. Al/Ga−P	2.41	0	2.43	0.62
Al/Ga−Al/Ga	3.05	0.66	3.10	0
Li−Li	2.89	0.35	2.88	1.95
Al/Ga−Li	2.92	1.85	2.92	0

## Conclusions

Li_3_AlP_2_ is a new representative of lithium phosphidoaluminates. It is the first lithium phosphidoaluminate with interconnected AlP_4_ tetrahedra. In the orthorhombic distorted lattice, the AlP_4_ tetrahedra are connected via edges and corners to give ${}_{\infty }{}^{2}\left[{TrP_{2}}^{3-}\right]$ 2D layers. The lithium atoms are located between and within these layers. Li_3_GaP_2_ represents the first lithium phosphidogallate. Both phases are easily accessible through ball milling of the elements and subsequent annealing and show thermal stability up to 700 °C. In the respective MAS‐NMR spectra all different positions can be assigned individually. Though both compounds are poor ion conductors, band structure calculations reveal that Li_3_AlP_2_ and Li_3_GaP_2_ are direct band gap semiconductors with band gaps of 3.1 and 2.8 eV, respectively. These results demonstrate that lithium phosphidotrielates can—depending on the content of Li_3_P—also exhibit structures with connected *Tr*P_4_ tetrahedra.

## Conflict of interest

The authors declare no conflict of interest.

## Supporting information

As a service to our authors and readers, this journal provides supporting information supplied by the authors. Such materials are peer reviewed and may be re‐organized for online delivery, but are not copy‐edited or typeset. Technical support issues arising from supporting information (other than missing files) should be addressed to the authors.

Supplementary
