# Supplementary material for: Synthesis, Structure, Solid‐State NMR Spectroscopy, and Electronic Structures of the Phosphidotrielates Li3AlP2 and Li3GaP2
Source: Chemistry. 2020 Apr 28;26(30):6812–9. doi: 10.1002/chem.202000482 (PMC7317418; doi:10.1002/chem.202000482)
Supplement: Supplementary file 1 — Supplementary [file CHEM-26-6812-s001.pdf]

Chemistry–A European Journal

Supporting Information

Synthesis, Structure, Solid-State NMR Spectroscopy, and Electronic Structures of the Phosphidotrielates Li_3AlP_2 and Li_3GaP_2

Tassilo M. F. Restle,[a] Jasmin V. Dums,[a] Gabriele Raudaschl-Sieber,[b] and Thomas F. Fässler*[a]

SUPPORTING INFORMATION

<u>Content:</u>

Powder X-ray diffraction patterns after ball milling

Color of the products

EDX measurement

Powder X-ray diffraction patterns of the element reaction

DSC measurement

Details on the crystal structure

Impedance spectroscopy

Details of the quantum-chemical calculations

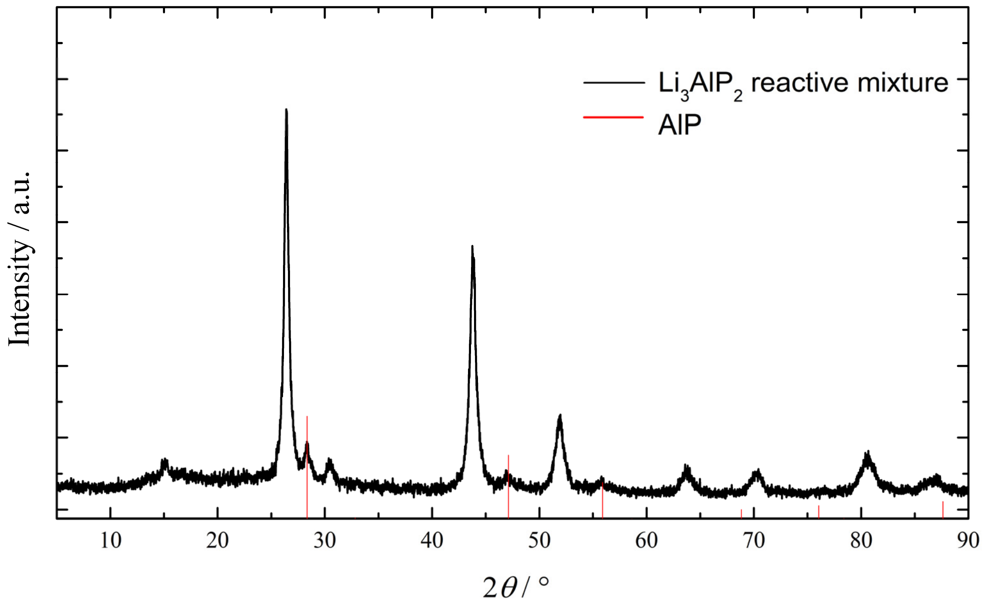

Figure S1. Experimental powder X-ray diffraction pattern of the product of ball milled elements in a stochiometric ratio of 3 Li + Al + 2 P.

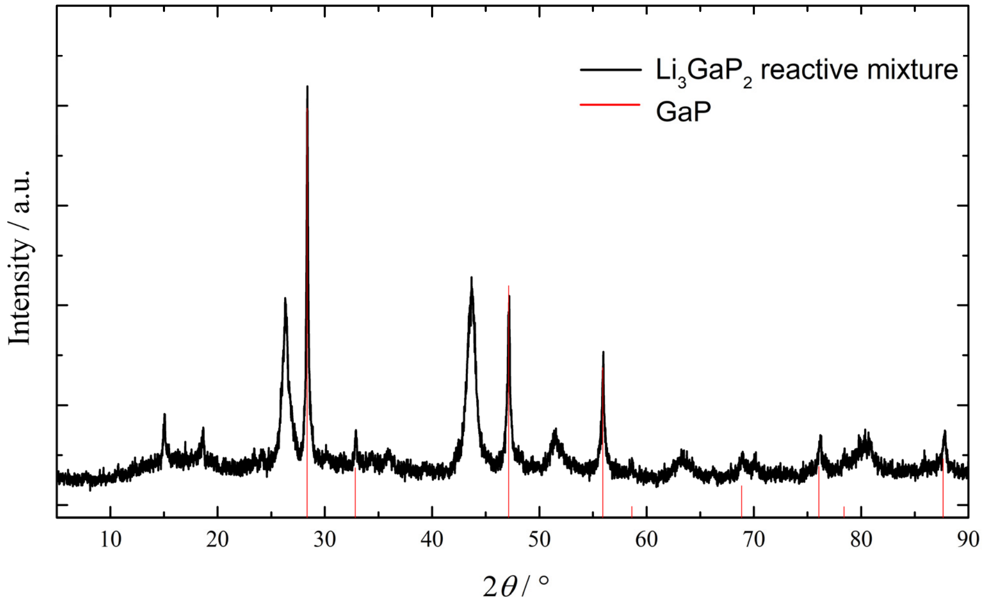

Figure S2. Experimental powder X-ray diffraction pattern of the product of ball milled elements in a stochiometric ratio of 3 Li + Ga + 2 P.

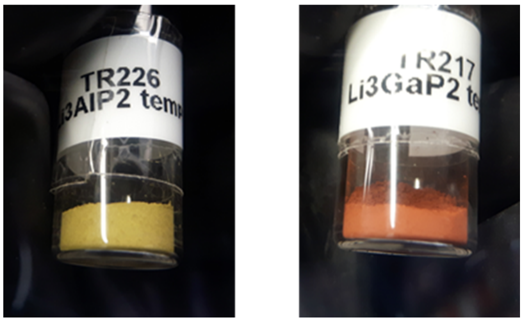

Figure S3. Pictures of the products obtained after ball milling and subsequently annealing. Left: yellow ochre Li_3AlP_2. Right: brick red Li_3GaP_2.

Table S1. Al/P and Ga/P ratios from EDX measurements for Li_3AlP_2 and Li_3GaP_2, respectively, in comparison with the theoretical ratio from the nominal compositions.

	$\%_{exp}$ Al	$\%_{exp}$ Ga	$\%_{exp}$ P	$\%_{theo}$ Al	$\%_{theo}$ Ga	$\%_{theo}$ P
**Li_3AlP_2**	33.7(5)	-	66.3(5)	33.3	-	66.7
**Li_3GaP_2**	33(2)		66(2)	-	33.3	66.7

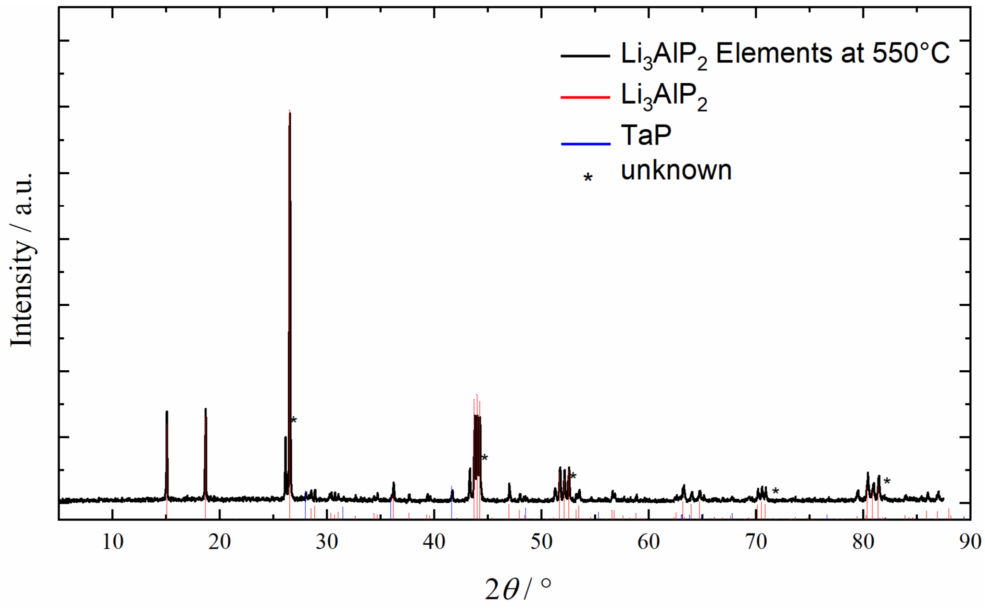

Figure S4. Experimental powder X-ray diffraction pattern of the product of the reaction 3 Li + Al + 2 P at 550 °C.

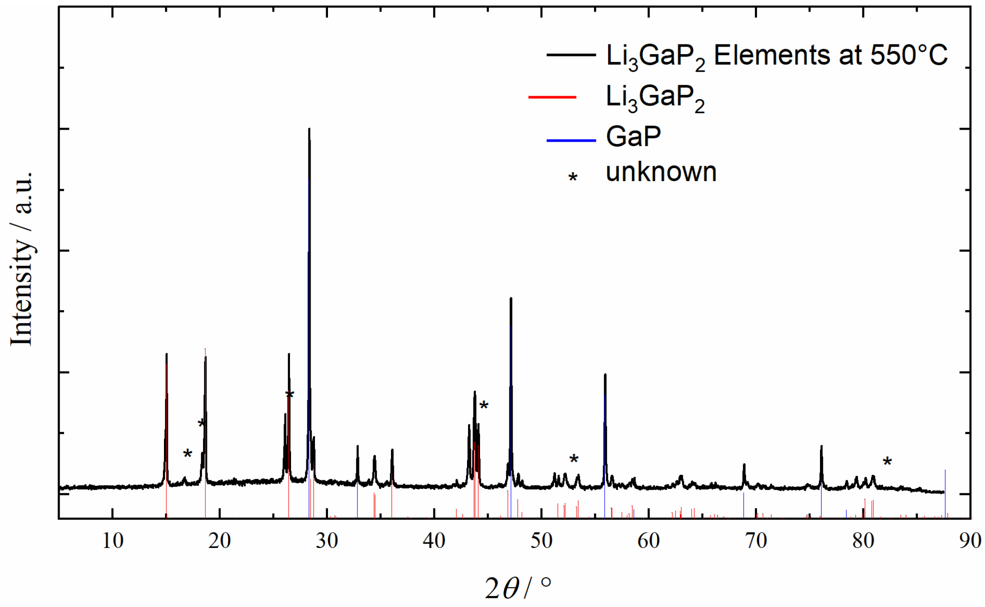

Figure S5. Experimental powder X-ray diffraction pattern of the product of the reaction 3 Li + Ga + 2 P at 550 °C.

Table S2. Atomic coordinates, isotropic displacement parameters (Å^2) and *Tr*-P bond lengths (Å) for Li_3AlP_2 and Li_3GaP_2 from Rietveld analyses.

		**Li_3AlP_2**				**Li_3GaP_2**			
Atom	Wyckh.	x	y	z	U_{iso} / Å^2	x	y	z	U_{iso} / Å^2
P1	8f	0	0.1175(3)	0.2138(3)	0.0167(8)	0	0.1164(2)	0.2117(3)	0.0251(3)
P2	8e	¼	0.3850(3)	¼	0.0196(9)	¼	0.3826(2)	¼	0.0247(9)
Al1	8d	0.1315(3)	0	0	0.0202(5)	0.1333(1)	0	0	0.0164(9)
Li1	8d	0.384(2)	0	0	0.018(1)	0.110(1)	0	½	0.012(3)
Li2	16g	0.123(2)	0.2429(5)	0.4805(1)	0.018(1)	0.117(1)	0.2439(6)	0.484(2)	0.036(2)
d(*Tr*1 – P1) / Å				2.398(3)				2.404(2)	
d(Tr1 – P2) / Å				2.410(3)				2.419(2)	

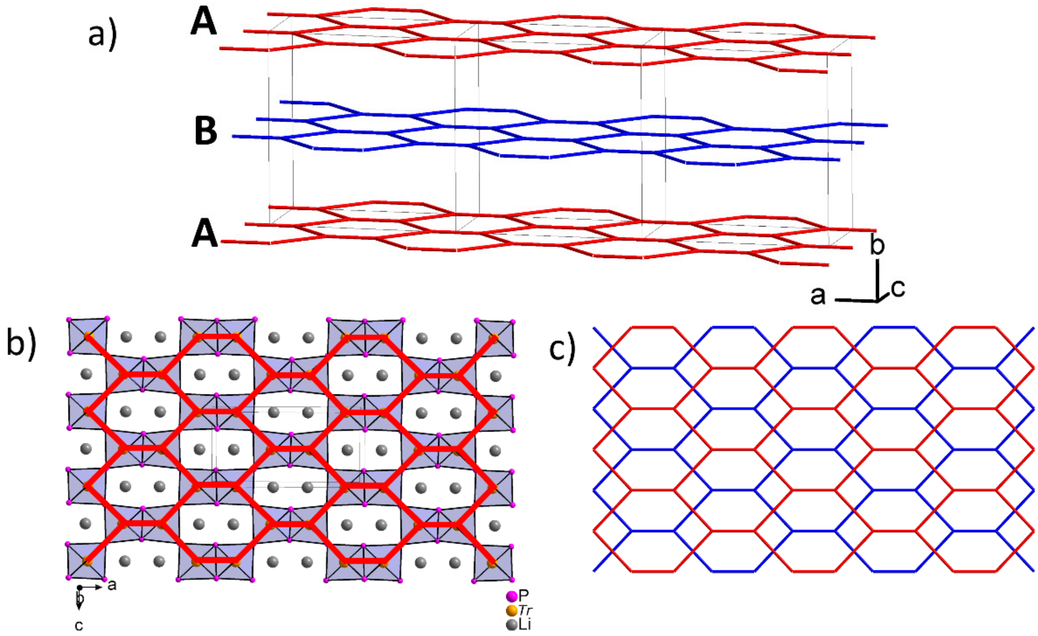

Figure S6. a) ABAB stacking sequence of the anionic 2D layer ${}^{2}_{\infty}[TrP_2{}^{3-}]$. The different layers A and B are highlighted in red and blue, respectively. For clearness, the atoms are not shown. Every point of intersections stands for one TrP_4 tetrahedron. b) View onto one layer in Li_3TrP_2 in *b* direction. In *a* direction the tetrahedra are connected by both, edge- and corner-sharing, whereas in *c* direction only corner-sharing occurs. c) View onto layers in Li_3TrP_2 in *b* direction.

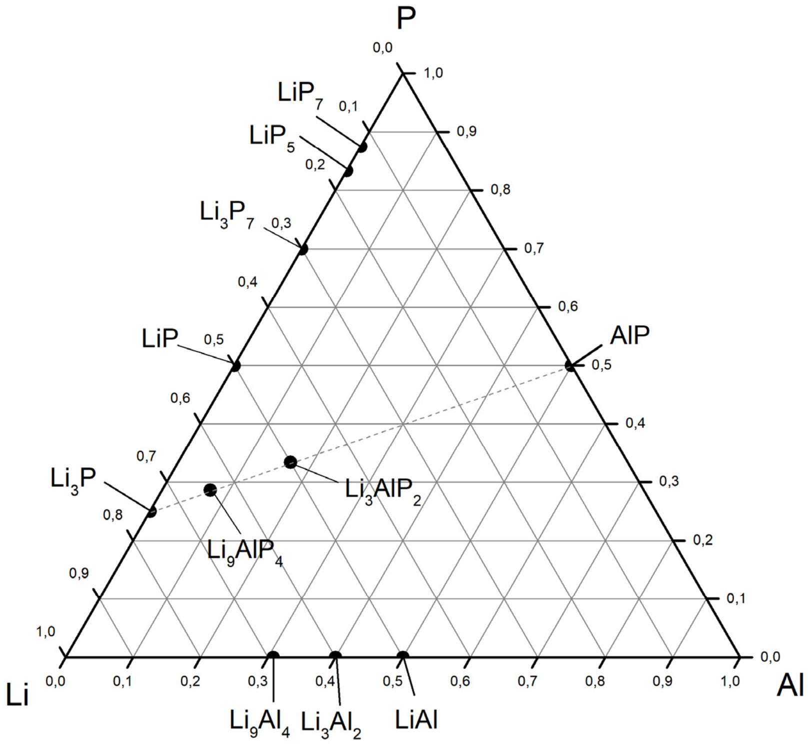

Figure S7. Ternary phase system of Li–Al–P.

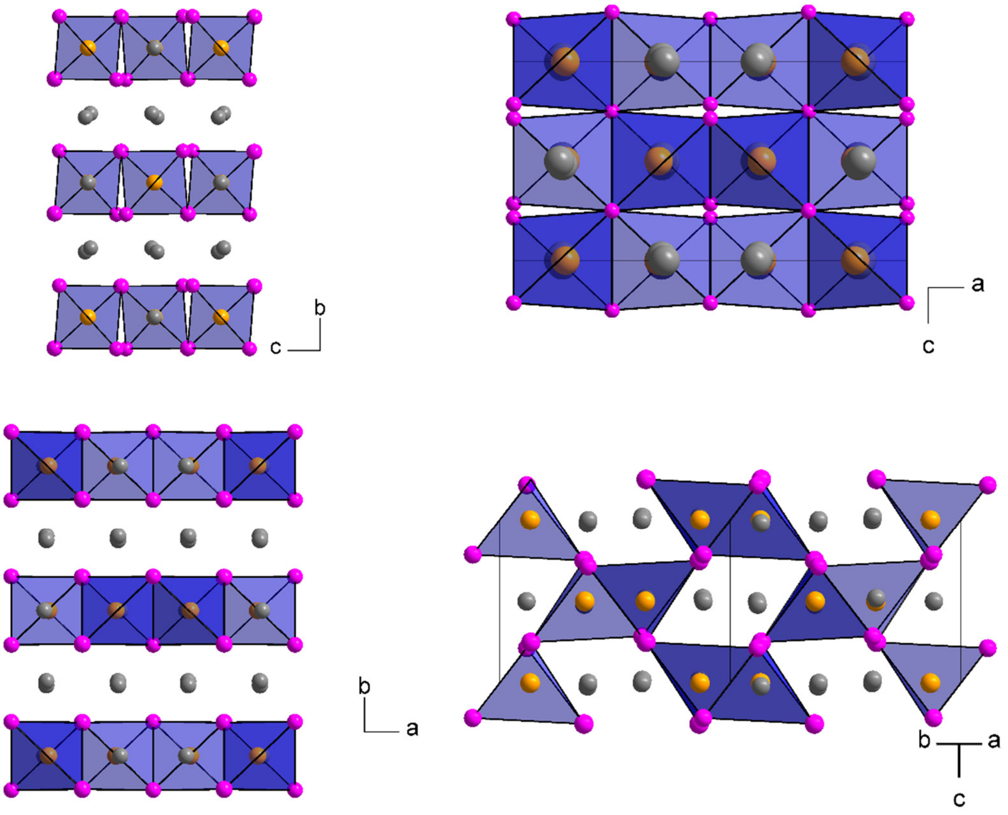

Figure S8. View in direction of the axes *a*, *b* and *c* of Li_3TrP_2 and in direction of the space diagonal. Only in *a* and *c* direction the lithium pathways are open, whereas in *b* direction the TrP_4 tetrahedra form blocking layers. Nevertheless, a 3D pathway is also open, namely in direction of the space diagonal.

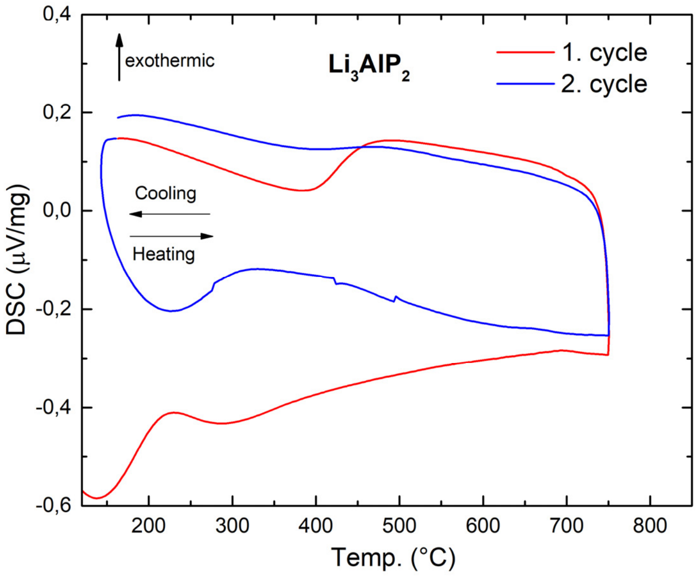

Figure S9. DSC curves of Li_3AlP_2. Two measurement cycles were performed using heating/cooling rates of 10 °C min^{-1}. The very sharp, small signals at around 400 °C and 500 °C are assigned to measurement artefacts. No significant signals arise during the two cycles. Hence, Li_3AlP_2 is stable up to 750 °C. PXRD analysis of the products after the DSC measurement proofs that the phase remains unchanged. (Fig S12).

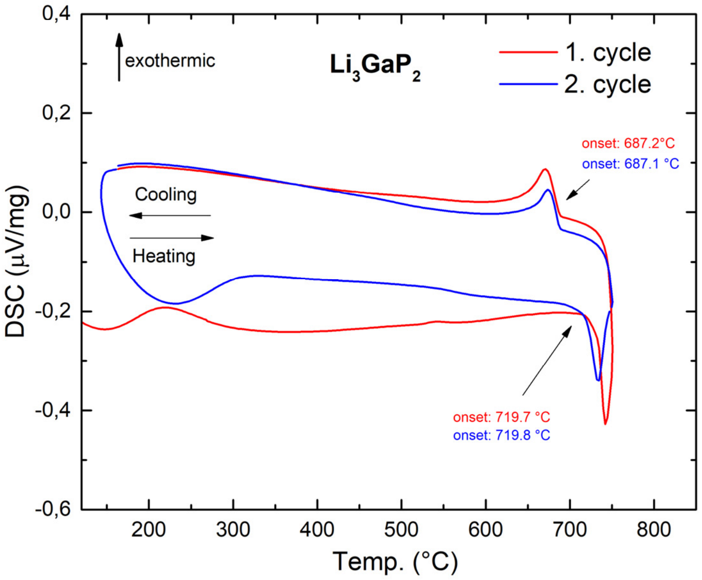

Figure S10. DSC curves of Li_3GaP_2. Two measurement cycles were performed using heating/cooling rates of 10 °C min^{-1}. One reversible signal arises in both cycles, at approximately 720 °C during heating and at 687 °C during cooling, assignable to a melting, phase change or decomposition process. PXRD analysis of the products after the DSC measurement proofs that the phase is still present, together with a small amount of an unknown side phase. (Fig S13). Hence, Li_3GaP_2 is only stable up to about 710 °C.

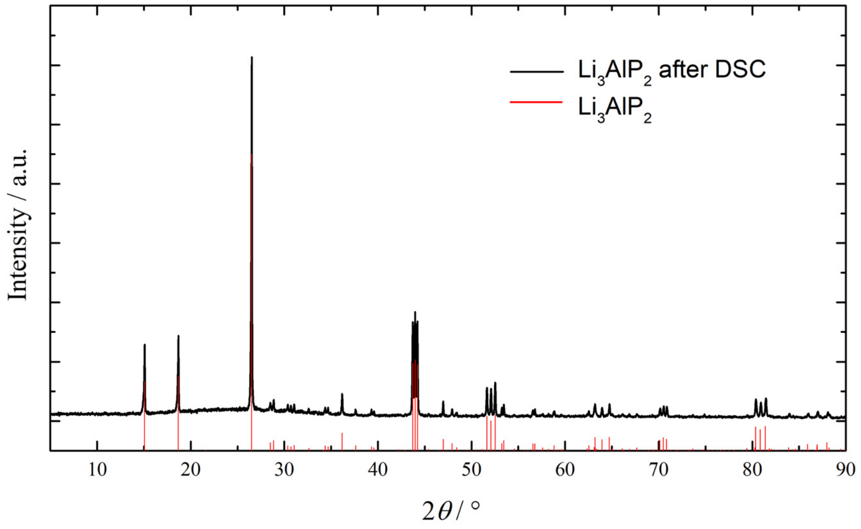

Figure S11. Experimental powder X-ray diffraction pattern of Li_3AlP_2 after the DSC measurement.

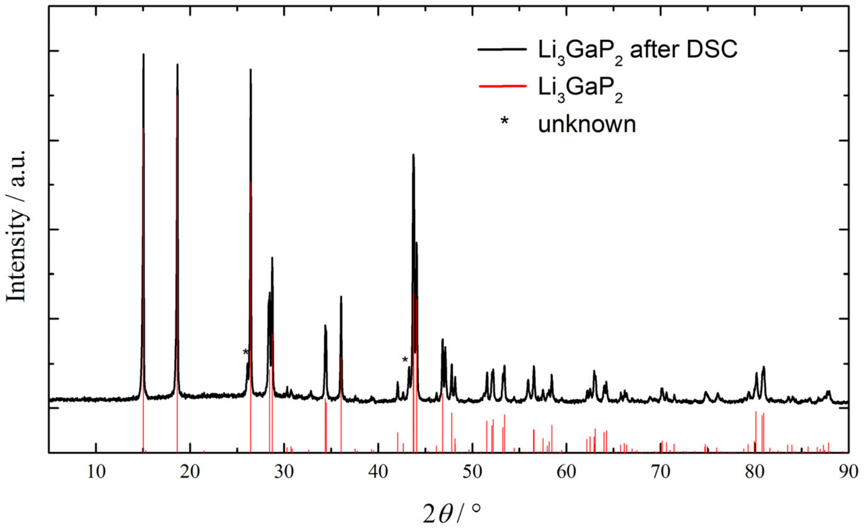

Figure S12. Experimental powder X-ray diffraction pattern of Li_3GaP_2 after the DSC measurement.

Table S3. Selected interatomic distances in Li_3AlP_2.

atom pair			*d* / Å	atom pair			*d* / Å
P1	Al1	2x	2.398(3)	Li1	P2	2x	2.54(1)
	Li1	2x	2.54(1)		P1	2x	2.56(1)
	Li2	2x	2.56(1)		Li1	1x	2.67(3)
	Li2	2x	2.57(1)		Li2	2x	2.861(6)
P2	Al1	2x	2.410(3)		Al1	3x	2.91(2)
	Li1	2x	2.52(1)	Li2	P1	1x	2.56(1)
	Li2	2x	2.59(1)		P1	1x	2.57(1)
	Li2	2x	2.62(1)		P2	1x	2.59(1)
Al1	P1	2x	2.398(3)		P2	1x	2.62(1)
	P2	2x	2.410(3)		Li2	1x	2.85(3)
	Li1	3x	2.91(2)		Li1	1x	2.861(6)
	Li2	2x	3.027(6)		Li2	3x	2.92(3)

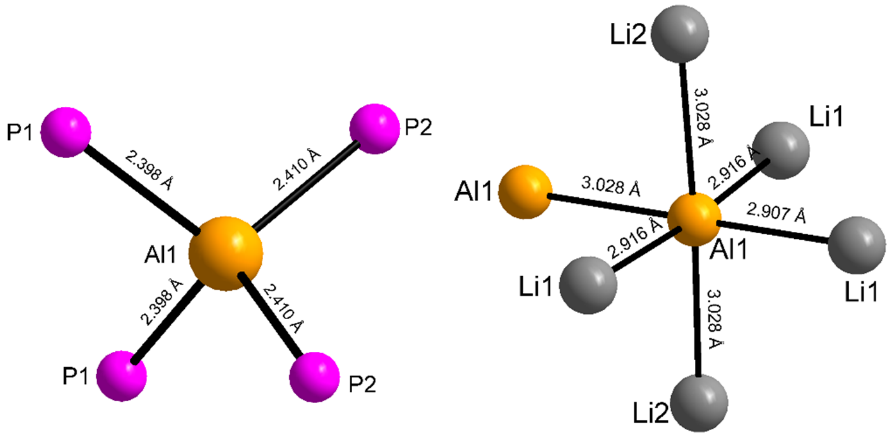

Figure S13. Coordination polyhedra of the Al atoms in Li_3AlP_2. In the first coordination sphere the neighbors form slightly distorted tetrahedra and in the second coordination sphere slightly distorted octahedra.

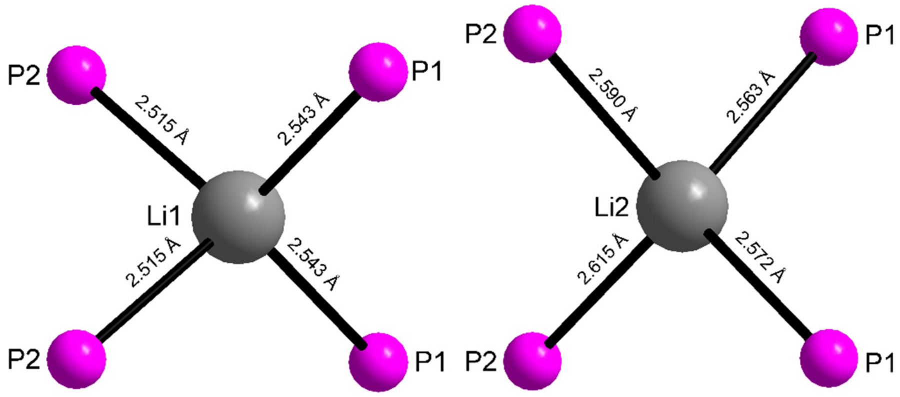

Figure S14. Coordination polyhedra of the Li atoms in Li_3AlP_2. The neighbors form slightly distorted tetrahedra.

Table S4. Selected interatomic distances in Li_3GaP_2.

atom pair			*d* / *Å*	atom pair			*d* / *Å*
P1	Ga1	2x	2.404(2)	Li1	P2	2x	2.515(6)
	Li2	2x	2.51(1)		Li1	1x	2.56(2)
	Li1	2x	2.515(6)		P2	2x	2.577(8)
	Li2	2x	2.57(1)		Li2	2x	2.876(7)
P2	Ga1	2x	2.419(2)		Ga1	2x	2.919(1)
	Li1	2x	2.577(8)		Ga1	1x	2.97(1)
	Li2	2x	2.63(1)	Li2	P1	1x	2.51(1)
	Li2	2x	2.65(1)		P1	1x	2.57(1)
Ga1	P1	2x	2.404(2)		P2	1x	2.63(1)
	P2	2x	2.419(2)		P2	1x	2.65(1)
	Li1	2x	2.919(1)		Li2	1x	2.70(2)
	Li1	1x	2.97(1)		Li1	1x	2.876(7)
	Li2	2x	3.024(7)		Li2	2x	2.91(2)

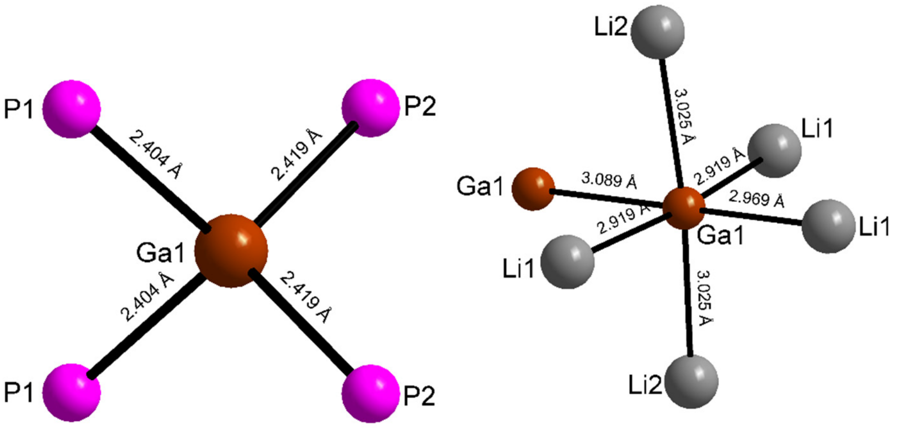

Figure S15. Coordination polyhedra of the Al atoms in Li_3GaP_2. In the first coordination sphere the neighbors form slightly distorted tetrahedra and in the second coordination sphere slightly distorted octahedra.

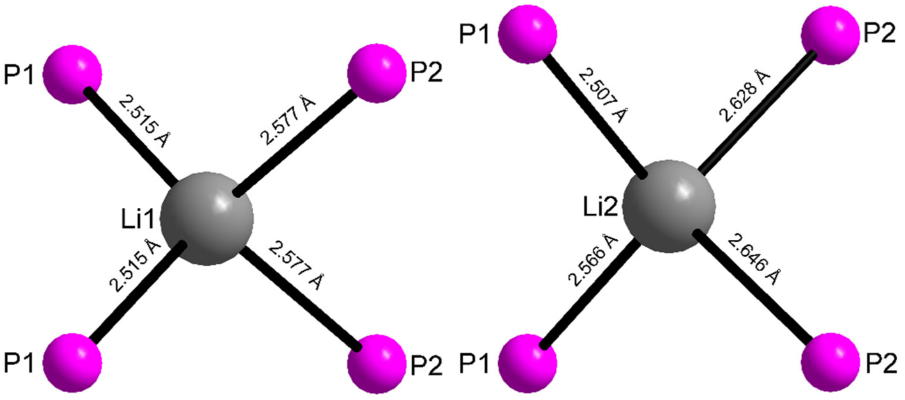

Figure S16. Coordination polyhedra of the Li atoms in Li_3GaP_2. The neighbors form slightly distorted tetrahedra.

Impedance Spectroscopy

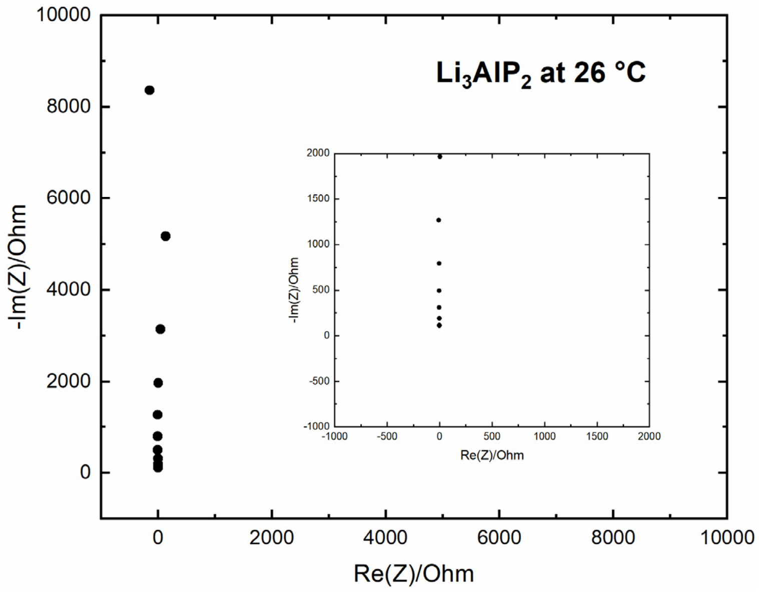

Figure S17. Nyquist plot of Li_3AlP_2 measured under blocking conditions, recorded at 26 °C.

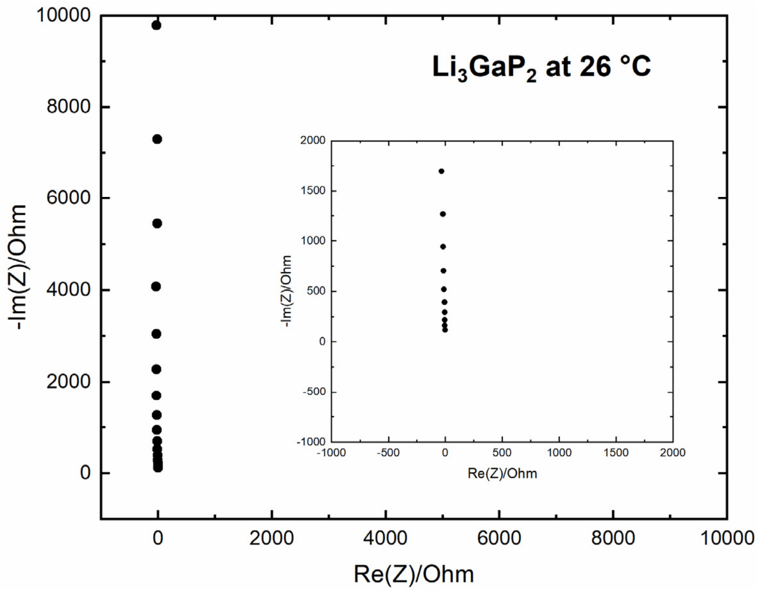

Figure S18. Nyquist plot of Li_3GaP_2 measured under blocking conditions, recorded at 26 °C.

Details of the quantum chemical calculations

Table S5. Frequencies of Li_3AlP_2 and Li_3GaP_2 from frequencies analysis at Γ-point**.**

**Li_3AlP_2 Frequencies / cm^{-1}**	**Li_3GaP_2 Frequencies / cm^{-1}**
0.00	0.00
0.00	0.00
0.00	0.00
142.07	109.16
147.88	109.30
149.67	113.22
174.80	126.55
177.90	131.73
178.65	133.36
186.85	139.05
189.89	157.38
196.59	169.06
197.45	172.94
206.75	177.02
214.79	183.49
219.36	187.28
228.38	192.42
228.89	195.82
235.23	195.90
240.91	215.50
241.00	222.37
245.10	235.56
246.71	237.65
262.03	243.12
267.50	243.83
272.17	250.57
273.53	251.52
274.77	257.00
276.02	266.26
287.53	276.04
296.69	276.49
298.20	277.14
302.17	279.40
314.26	283.18
316.07	292.24
317.99	299.59
320.20	301.44
324.37	304.38
338.11	313.93
339.37	314.05
341.05	316.64
348.10	318.27
350.36	320.13
352.72	326.17
355.45	332.75
356.73	334.18
359.79	335.67
364.06	346.39
373.24	347.85
373.90	352.01
374.26	352.86
377.77	353.81

380.12	356.83
387.30	359.15
387.63	363.91
389.88	366.22
396.10	372.45
396.64	385.32
404.85	389.29
404.86	389.49
409.69	393.34
410.97	399.52
417.90	403.68
420.42	406.89
434.37	411.06
443.13	420.88
458.53	422.46
463.46	428.41
471.47	445.39
490.72	447.45
502.44	454.71
504.92	466.22

Text S-1. Detailed description of the used basis sets and basis set listings in CRYSTAL format.

Li: The diffuse outermost s-exponents in def-SVP were increased from 0.021 and 0.053 to 0.18 and 0.36, respectively[F. Weigend, R. Ahlrichs; *Phys. Chem. Chem. Phys.* **2005**, 7, 3297., A.Karttunen, private communication] The (2*p*)/[1*p*] polarization function with exponents of 0.45 and 0.1 was replaced by one primitive p-type polarization function with an exponent of 0.18 (which is close to the exponent of 0.17 in the "Pold" polarization function of TURBOMOLE library. The outermost *s* and *p* functions were finally combined into a single *sp*-type function.

```
3 3
0 0 5 2.0 1.0
  266.27785516      0.64920150325E-02
  40.069783447      0.47747863215E-01
  9.0559944389      0.20268796111
  2.4503009051      0.48606574817
 0.72209571855      0.43626977955
0 0 1 1.0 1.0
 0.36               1.0000000000
0 1 1 0.0 1.0
 0.18               1.0 1.0
```

P: For the triple-zeta-valence basis set, the def-TZVP basis set was used as a starting point[F. Weigend, R. Ahlrichs; *Phys. Chem. Chem. Phys.* **2005**, 7, 3297., A.Karttunen, private communication] We fixed the exponents of the outermost *s* and *p* functions to 0.13 and reoptimized the exponents of the other *s* and *p* functions in the valence space for the phosphorus atom in its ground state. Finally, the outermost *s* and *p* functions were combined into *sp*-type functions. The resulting energy loss with respect to the original molecular basis set is 2.9 mH. We applied the original *d*-polarization function with an exponent of 0.45.

```
15 8
0 0 7 2.0 1.0
  52426.999233      0.55207164100E-03
  7863.2660552      0.42678595308E-02
  1789.5227333      0.21931529186E-01
  506.27300165      0.85667168373E-01
  164.60698546      0.24840686605
  58.391918722      0.46336753971
  21.643663201      0.35350558156
0 0 3 2.0 1.0
  99.013837620      0.21895679958E-01
  30.550439817      0.95650470295E-01
  5.4537087661     -0.29454270186
0 0 2 2.0 1.0
  2.6477257457       1.3294381200
  1.2738231734      0.66109396473
0 1 1 0.0 1.0
```

```
  0.35000000000      1.0 1.0
0 1 1 0.0 1.0
  0.13000000000      1.0 1.0
0 2 6 6.0 1.0
  472.27219248       0.25710623052E-02
  111.58882756       0.20250297999E-01
  35.445936418       0.91580716787E-01
  12.990776875       0.25749454014
  5.0486221658       0.42862899758
  1.9934049566       0.34359817849
0 2 1 3.0 1.0
 0.69644412108       1.00000000000
0 3 1 0.0 1.0
  0.450              1.0
```

Al: The def-TZVP basis set was used as a starting point[F. Weigend, R. Ahlrichs; *Phys. Chem. Chem. Phys.* **2005**, 7, 3297., A.Karttunen, private communication]. We fixed the exponents of the outermost *s* and *p* functions to 0.10 and reoptimized the exponents of the other *s* and *p* functions in the valence space for the aluminum atom in its ground state. Finally, the outermost *s* and *p* functions were combined into *sp*-type functions. The resulting energy loss with respect to the original molecular basis set is 8.6 mH. The original *d*-polarization function with an exponent of 0.30 was applied.

```
13 9
0 0 7 2.0 1.0
  37792.550772       0.57047888709E-03
  5668.0682165       0.44093016538E-02
  1289.8582841       0.22630967411E-01
  364.86596028       0.88025644295E-01
  118.57631515       0.25223701612
  42.024867605       0.45960547169
  15.499501629       0.33277886014
0 0 3 2.0 1.0
  75.208026598       0.19250560190E-01
  23.031408972       0.87906743952E-01
  3.6348797649      -0.34246704535
0 0 2 2.0 1.0
  1.6020065437        1.5106266058
 0.78069416556       0.58071016470
0 0 1 0.0 1.0
  0.27777633704      1.0
0 1 1 0.0 1.0
  0.10000000000      1.0 1.0
0 2 6 6.0 1.0
  452.52303192       0.23110812466E-02
  107.08195049       0.18568641823E-01
  34.131021255       0.87216237035E-01
  12.587037428       0.26902101523
  4.9811919704       0.52128324272
  2.0070350900       0.60271687494
0 2 1 1.0 1.0
 0.82081434825       1.0
0 2 1 0.0 1.0
 0.39036158090       1.0
0 3 1 0.0 1.0
  0.300              1.0
```

Ga: The def-TZVP basis set was used as a starting point.[F. Weigend, R. Ahlrichs; *Phys. Chem. Chem. Phys.* **2005**, 7, 3297., A.Karttunen, private communication] We fixed the exponents of the outermost *s* and *p* functions to 0.09 and reoptimized the exponents of the other *s* and *p* functions in the valence space for the gallium atom in its ground state. Finally, the outermost s and p functions were combined into *sp*-type functions. At this point, the resulting energy loss with respect to the original molecular def-TZVP basis set was 3.0 mH. Next, we kept the outermost *d*-function fixed and optimized the exponents of the other *d*-functions in the valence space, analogously to the derivation of molecular def2-TZVP basis set for In–I and Tl–At.[F. Weigend, R. Ahlrichs; *Phys. Chem. Chem. Phys.* **2005**, 7, 3297.] The energy was improved and the final energy was is 1.5 mH lower than in the molecular def-TZVP basis set.

```
31 14
0 0 8 2.0 1.0
  435548.66254       0.23646329650E-03
  65289.589031       0.18335271776E-02
  14858.784256       0.95371863081E-02
  4205.9734729       0.38803412468E-01
  1369.6416431       0.12661604848
  492.30348905       0.30175310292
  191.41923233       0.43543934218
```

```
  75.840558665      0.23282363780
0 0 4 2.0 1.0
  474.30810613     -0.26743707958E-01
  147.10297560     -0.12654657542
  23.982599435      0.58840346839
  10.298230094      0.56324271589
0 0 2 2.0 1.0
  16.050381430     -0.24516439508
  2.6988468784      0.74578049593
0 0 1 2.0 1.0
  1.1452841362       1.0000000000
0 0 1 0.0 1.0
 0.25080935147       1.0000000000
0 1 1 0.0 1.0
 0.09000000000       1.0 1.0
0 2 6 6.0 1.0
  2432.0171070      0.22434065928E-02
  576.12049582      0.18342265336E-01
  185.11584354      0.87279697167E-01
  69.246572556      0.25684868351
  27.818107777      0.42398378107
  11.420229938      0.25701340043
0 2 3 6.0 1.0
  42.819661530     -0.19326519119E-01
  6.3885901000      0.31571386917
  2.6698993326      0.57617792822
0 2 1 1.0 1.0
 1.0965804072        1.0000000000
0 2 1 0.0 1.0
 0.32984082243       1.0000000000
0 3 5 10.0 1.0
  107.38114308      0.11464613652E-01
  31.543551222      0.73625747383E-01
  11.422960020      0.23505107382
  4.4828352515      0.40318563513
  1.7778113384      0.40824748152
0 3 1 0.0 1.0
 0.67759049087       1.0000000000
0 3 1 0.0 1.0
 0.20700000000       1.0000000000
0 4 1 0.0 1.0
 0.30996095          1.0
```
